# Improving Pool Fencing Legislation in Queensland, Australia: Attitudes and Impact on Child Drowning Fatalities

**DOI:** 10.3390/ijerph14121450

**Published:** 2017-11-24

**Authors:** Richard C. Franklin, Amy E. Peden

**Affiliations:** 1 College of Public Health, Medical and Veterinary Sciences, James Cook University, Townsville, QLD 4811, Australia; 2 Royal Life Saving Society—Australia, Broadway, NSW 2007, Australia; apeden@rlssa.org.au

**Keywords:** drowning prevention, child drowning, injury prevention, engineering, pool fencing, epidemiology, hierarchy of controls, social ecological model, health promotion

## Abstract

Four-sided, non-climbable pool fencing is an effective strategy for preventing children from drowning in home swimming pools. In 2009, the Queensland Government introduced legislation to improve the effectiveness of pool fencing. This study explores community attitudes towards the effectiveness of these legislative changes and examines child (<5 years) drowning deaths in pools. Data from the 2011 Queensland Computer-Assisted Telephone Interviewing (CATI) Social Survey include results from questions related to pool ownership and pool fencing legislation. Fatal child drowning cases between 1 January 2005 and 31 December 2015 were sourced from coronial data. Of the 1263 respondents, 26/100 households had a pool. A total of 58% believed tightening legislation would be effective in reducing child drowning deaths. Pool owners were more likely to doubt the effectiveness of legislation (*p* < 0.001) when compared to non-pool owners. Perceptions of effectiveness did not differ by presence of children under the age of five. There were 46 children who drowned in Queensland home pools (7.8/100,000 pools with children residing in the residence/annum) between 2005 and 2015. While pool owners were less likely to think that tightening the legislation would be effective, the number of children drowning in home swimming pools declined over the study period. Drowning prevention agencies have more work to do to ensure that the most vulnerable (young children in houses with swimming pools) are protected.

## 1. Introduction

Pool fencing should be considered one of the great injury prevention initiatives. At the height of the home pool drowning epidemic in Australia in the 1970s, seven children were drowning per annum in Brisbane (a city in the state of Queensland) [1], compared with nine children in the whole of Australia during the 2015–2016 financial year [2]. This simple yet effective solution [3] has taken many years to be implemented across Australia, and changes to legislation continue in each jurisdiction to ensure that it is as effective as possible [4].

**Figure 1:**
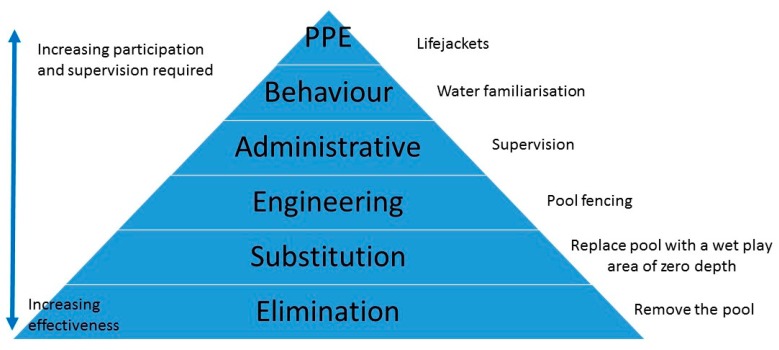
The Hierarchy of Controls and swimming pool safety.

Pool fencing fits into the engineering category of the Hierarchy of Controls; a six-domain pyramid that provides a framework for thinking about the effectiveness of a prevention strategy [5,6] (Figure 1). The Hierarchy of Controls is informed by the work of Dr. William Haddon, Jr., who also created the Haddon’s Matrix, a tool for investigating injury events to identify prevention strategies, originally developed to help understand vehicle crashes [5,7]. Dr. Haddon was also a major supporter of initiatives that removed the need for human intervention on an ongoing basis [7].

There are key elements that are required for a pool barrier to be effective. These include ensuring that fencing is present; that the gate self-closes and self-latches; that there are no gaps under the fence; that the fence is in good working order; and is of a height and design, which stops children from readily climbing the fence [8]. The mean age of children who drown in swimming pools from unintended access (i.e., are not already in the pool area) is between 2.0 years (SD = 0.88) [9] and 2.3 years [10], thus fencing has been predominantly targeted at children under the age of five years. 

**Figure 2:**
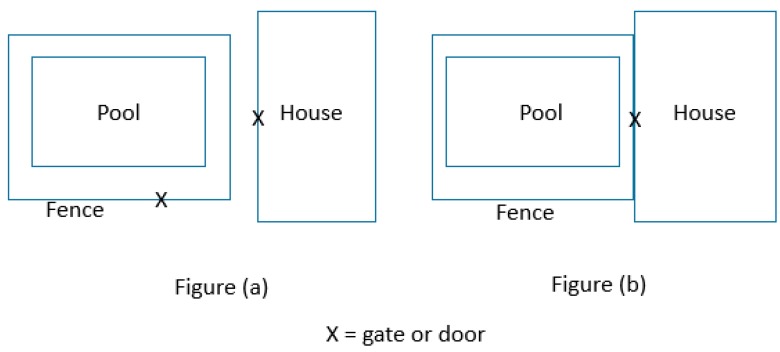
(**a**) Four-sided pool fencing; (**b**) Three-sided pool fencing modified from Australian Standard AS1926.2-2007 [14].

Evidence continues to be developed around both the effectiveness of pool fencing, and the elements which make pool fencing effective. This includes the configuration of the fence: originally a boundary fence was allowed, and over time this has moved to a three-sided pool fence and then a four-sided pool fence (Figure 2). Pitt and Balanda (1991) [11] found that four-sided fencing was twice as effective as three-sided fencing, which intuitively makes sense as pools with four-sided fencing are harder for the child to access and gives the parent more time to respond. The self-closing and self-latching gate has been an integral part of the fence for many years and ensures the integrity of the pool fence. The gate has been a common area of weakness [12,13] and the innovation of a magnetic latch which ensures the gate closes and latches has been a major boost to the effectiveness of the self-latching mechanism. 

Fence height and design continues to be a challenge, particularly in defining what an effective height is to stop children from climbing into the pool area, as well as ensuring that the fence is non-climbable. Early work by Nixon et al. (1979) found that a 1.4 m fence was able to be climbed by 0% of three-year-old children, a 1.2 m fence by 18%, and a 90 cm fence by 55% [15]. The preferred height was 1.4 m, however, a compromise was reached by the Australia Standards committee that 1.2 m would be used. Anecdotally, this was due to 1.2 m being a common height of widely available fencing material. Defining how climbable a fence is continues to be a challenge, however, most agree that it is the placement of horizontal elements of the fence that make it climbable or the presence of materials such as plants and outdoor furniture close to the fence with a surface allowing for food holds which make a fence easier to climb [16].

Like all built material, pool fences and surrounds deteriorate over time. The actual lifetime of a pool fence is highly variable and influenced by a range of factors including: local climate and weather; type and quality of material used; amount of use; local soils; and quality of workmanship.

In 2009, the Queensland Government introduced revised pool fencing laws for all new and existing home swimming pools. For new pools, the legislation came into effect on 1 December 2009 and then, a year later, existing pools were also included.

The 2009 legislation was aimed at addressing issues around: the climbability of the fence (through the introduction of a non-climbable zone), ensuring new swimming pools undergo final inspections and that existing pools are brought up to the new standard at the same time, addressing ongoing maintenance issues via a regular (five-year) inspection program, ensuring there is a cardiopulmonary resuscitation (CPR) sign, and addressing issues around fencing materials including where the pool fence intersects with other barriers such as retaining walls, other fences, and permanent bodies of water [17].

In 2010, all existing legislation was replaced with one pool safety standard, and all exemptions (apart from disability exemptions) were abolished [17]. For further information regarding the 2009 home swimming pool legislation changes, please visit (http://www.hpw.qld.gov.au/construction/BuildingPlumbing/PoolSafety/PoolSafetyLaws/Pages/default.aspx).

To help understand what influences people and determines attitudes and behaviours, the socio-ecological model has been proposed. This model takes into account environmental, individual, social, cultural, political, and economic factors to help process and then address these influences to help improve health or in the case of pool fencing, safety [18]. Understanding what influences people to improve their pool fencing will be critical to ensuring the safety of all children and the development of effective campaigns to drive improvements in child drowning statistics. To date, little work has explored this space in order to provide better evidence for the development of drowning prevention interventions.

This paper explores community attitudes around the perceived effectiveness of the legislative changes and changes to the number of child drowning deaths in pools before and after implementation of the legislation.

## 2. Materials and Methods

This paper uses two methods: a CATI (Computer Assisted Telephone Instrument) survey, and the collation and analysis of data derived from the Royal Life Saving Society—Australia (RLSSA) National Fatal Drowning Database (the Database).

### 2.1. 2011 CATI Survey

The Queensland Social Survey (QSS) is an annual state-wide omnibus style survey of households in the Australian state of Queensland. The survey uses CATI methodology and surveys a random state sample. For the purposes of data collection, Queensland was divided into two subdivisions for telephone interviewing, South East Queensland (SEQ) (around the Brisbane and Moreton areas, Australia) and the remainder of the state. The survey used a two-stage selection process, firstly selecting the households and secondly selecting respondent gender within the household. A minimum sample size of 400 or more for each subdivision was deemed suitable to give the survey enough power to compare the subdivisions [19].

The target population was all persons 18 years of age or older, who at the time of the survey were living in a dwelling unit in Queensland that could be contacted by direct-dialed, land-based telephone service. A random selection approach was used through a telephone database of randomly generated numbers which were selected using postcode (zip-code) parameters. Within the household, one eligible person was selected (based on gender) as the respondent for the interview [19].

The survey instrument consisted of three components: (1) a standardised introduction; (2) questions which reflected the specific research interests of the University and community researchers participating in the study (pool ownership and attitudes towards pool fencing questions were asked in this component of the survey); and (3) demographic questions. The survey is piloted prior to the full roll out and sponsored questions (such as the pool fencing questions) are tested for comprehension and categories of response.

The response rate to the survey, calculated by dividing the number of people participating in the survey either with a completed or partially completed interview by the number of people selected in the sample, was 31.9%. The estimated sampling error, at the 95% confidence level, for the Other Queensland area sample of 428 households and a 50/50 binomial percentage distribution is ±4.7%. Responses were provided to researchers as an SPSS file.

A total of 1265 people responded to the survey in 2011. Two respondents did not answer the question regarding pool ownership (don’t know, no response). As such, these two responses were removed from the CATI survey dataset, leaving 1263 cases for analysis. With respect to the 2009 home swimming pool legislative changes, respondents were asked ‘How effective do you think that tightening the pool fencing legislation will be in reducing child drowning deaths?’ Possible responses were: ‘very effective’, ‘effective’, ‘neither effective nor ineffective’, ‘very ineffective’, ‘don’t know’, and ‘no response’. For ease of analysis, ‘very effective’ and ‘effective’ were re-coded as ‘effective’; ‘neither effective nor ineffective’ and ‘very ineffective’ were recoded as ineffective and ‘don’t know’ and ‘no response’ were recoded as ‘no response’. 

Each respondent represents a household. The number of swimming pools in Queensland was calculated by using the proportion of positive response to having a swimming pool (25.7%) at their residence against the total number of residences in Queensland (1,826,449 private dwellings); this equals 468,543 swimming pools [20].

### 2.2. Royal Life Saving National Fatal Drowning Database (the Database)

Unintentional fatal drowning deaths among children aged 0–4 years (i.e., under five) in home swimming pools in Queensland for the 11 years between 2005 and 2015 were sourced from the Royal Life Saving Society—Australia National Fatal Drowning Database (the Database). The Database draws information from the National Coronial Information System (NCIS), year-round media monitoring, and reports from police. Information on child drowning cases in Queensland are supplemented through the Queensland Family and Child Commission (QFCC). The method for sourcing drowning data from the NCIS and the Database has been published previously [21]. 

Cases within the coronial system remain open while they are under investigation by a coroner. A case is closed when a coroner makes a ruling on cause of death and/or takes the case to coronial inquest, where coronial recommendations may be made to prevent future similar deaths. Data within this study is correct as of 19 July 2017. At this time, 100% of cases in this study were closed.

The remoteness classification of the postcode of the drowning incident location was determined by using the Doctor Locator website [22]. Remoteness classifications fall into five categories: major cities, inner regional, outer regional, remote, and very remote [23].

Age-specific drowning rates per 100,000 population were calculated using population data for Queensland from the Australian Bureau of Statistics [24]. Rates of child drowning per 10,000 private swimming pools in Queensland were calculated using the estimate from the survey at the mid-point of the drowning data. 

A home swimming pool was defined as a swimming pool (either in-ground or above ground) in private residential premises or a portable pool (capable of being filled to a depth of 30 cm or more without filtration, and capable of being emptied and moved around) [25]. The definition includes outdoor spas (as they are subject to the same fencing legislation as home and portable pools), however there were no drowning deaths in outdoor spas of children 0–4 years in Queensland during the period of this study.

The season of drowning incident in Australia is defined as Summer (December, January, February), Autumn (March, April, May), Winter (June, July, August), and Spring (September, October, November). Time of day of drowning incident was classified into four time bands: Morning (6:01 a.m. to 12:00 p.m.), afternoon (12:01 p.m. to 6:00 p.m.), evening (6:01 p.m. to 12:00 a.m.), and early morning (12:01 a.m. to 6:00 a.m.).

Information on the child’s point of entry to the pool was drawn from the coronial finding and the police report. This information was coded into a new variable using the following categories: child gained access by climbing the fence; child gained access through a weakness in the fence; child gained access as the gate did not latch correctly; child gained access through a gate propped open; or child was already in the pool area.

### 2.3. Data Analysis

Data was analysed using SPSS V20 (SPSS Inc., Chicago, IL, USA) [26]. Chi square analysis was calculated with a 95% confidence interval. Where chi square analysis was undertaken with variables (e.g., age groups, remoteness, season etc.) where degrees of freedom was greater than one and were found to be initially statistically significant, a chi square analysis of the categories within the variable were then undertaken to explore where the statistically significant results occurred. All analysis was performed without the ‘unknown’ or ‘don’t know/no response’ variables. A modified Bonferroni, as suggested by Keppel [27] has been applied, deeming statistical significance *p* < 0.01.

For the drowning deaths over time, a linear trend line was undertaken in Excel. When comparing changes over time to drowning deaths pre and post the legislative changes, the year 2010 was used to separate the time before and after the 2009 legislative changes, as they came into effect on 1 December 2009 for new pools and 1 December 2010 for existing pools. Therefore, the year 2010 has been removed from the chi square analysis comparing child drowning deaths in home swimming pools in Queensland in the five years pre and five years post implementation of the legislation.

For the results of the CATI survey, a binary logistic regression was undertaken using SPSS based on a dichotomous variable of pool fencing effectiveness yes/no. For the CATI survey results, analysis of variance was undertaken for the purposes of determining the relative difference in perceived effectiveness, using SPSS. We have assumed the difference between each of the categories is linear for this purpose.

### 2.4. Ethics Approval

The drowning data collection was approved by the Department of Justice and Regulation Human Research Ethics Committee (JHREC) (CF/07/13729; CF/10/25057, CF/13/19798). The 2011 CATI survey received approval by the Human Research Ethics Review Panel at CQUniversity before administration to the general public (H10/06-121).

## 3. Results

### 3.1. CATI Survey

#### 3.1.1. Home Pool Ownership Chi Square Analysis

A total of 1263 people (50.0% female) were surveyed. Of these, almost two-thirds (66.2%) were from SEQ. People aged 55+ represented the largest age group (50.3%), with three-quarters (77.6%) of respondents born in Australia. One-quarter of all respondents (25.7%) had a pool at their place of residence. Being aged 45–54 years (X^2^(1) = 8.99; *p* < 0.01), having been educated for 1–10 years (X^2^(1) = 25.12; *p* < 0.001), a household income of between $26,001 and $52,000 (X^2^(1) = 13.21; *p* < 0.001) or greater than $100,000 (X^2^(1) = 34.34; *p* < 0.001), a household with a child under 18 (X^2^(1) = 19.48; *p* < 0.001), those who were married or de facto (X^2^(1) = 10.26; *p* = 0.001), and home ownership (X^2^(1) = 34.13; *p* < 0.001) were significant indicators that a respondent was more likely to have a home pool (Table 1).

#### 3.1.2. Home Pool Ownership Logistic Regression

The logistic regression (based on Table 1) found that those who own their home were 2.9 times more likely to have a home pool (*p* < 0.01; 95% CI: 1.5–5.4). Compared to 18–34 year olds, 35–44 years were 2.2 times (*p* < 0.05 95% CI: 1.2–4.0), 45–54 years were 3.3 times (*p* < 0.01; 95% CI: 1.9–5.5), and 55+ years were 2.0 times (*p* < 0.01; 95% CI: 1.4–3.1) more likely to have a home swimming pool.

#### 3.1.3. Pool Fencing Legislation Effectiveness Chi Square and Logistic Regression Analysis

When examining attitudes toward the effectiveness of changes to pool fencing legislation on reducing child drowning deaths, 57.5% of respondents felt the legislation changes would be effective. Those who believed changes to pool fencing legislation would be effective were commonly female (60.4% of females responded positively for effectiveness), aged 18–34 years (62.3%), born in Australia (58.3%), those who had been educated for 1–10 years (61.6%), and with a household income of $26,000 or less (66.2%) (Table 2).

People who listed their marital status as single, widowed, divorced, or separated were significantly more likely to believe changes to pool fencing legislation would be effective (X^2^(1) = 10.56; *p* = 0.001). Those who own their home (X^2^(1) = 7.51; *p* < 0.01) and those with home swimming pools (X^2^(1) = 32.25; *p* < 0.001) were significantly less likely to think the changes to pool fencing legislation would be effective in reducing child drowning deaths. There was no statistically significant difference when the logistic regression was applied (Table 2).

Almost three percent (2.9%) of respondents had both a home pool and children under the age of five in the household. Of these, 48.6% thought changes to pool fencing legislation would be effective in reducing drowning deaths in children under the age of five.

#### 3.1.4. Home Pool Legislation Effectiveness ANOVA Analysis

**Figure 3:**
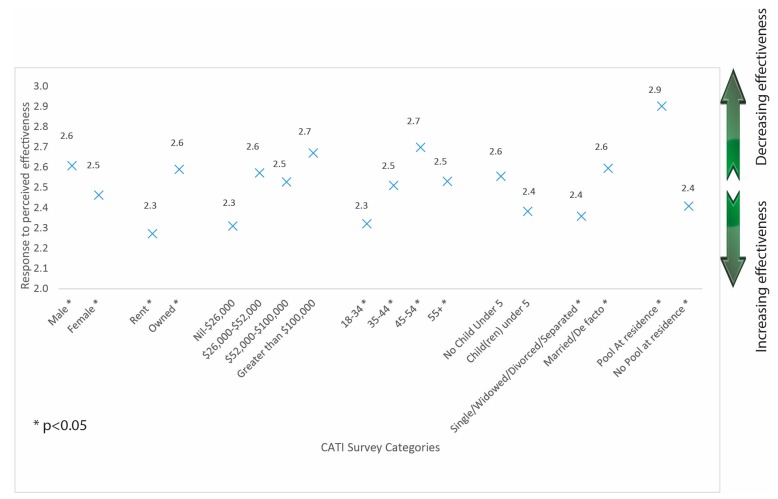
Perceived effectiveness of changes in Queensland to pool fencing legislation to achieve reduction in child drowning deaths by CATI survey categories.

An ANOVA was used to compare the perceived likelihood of the effectiveness of the legislation changes by category. As above, people with pools at their residence were significantly (F(1, 1224) = 36.1; *p* < 0.01) less likely to think the changes to pool fencing legislation would be effective in reducing child drowning deaths. Females (F(1, 1224) = 4.0; *p* < 0.05), people who rented (F(1, 1209) = 10.5; *p* < 0.01), younger people (18–34 years) (F(4, 1224) = 2.9; *p* < 0.05), and those who reported being single, widowed, divorced or separated (F(1, 1222) = 8.1; *p* < 0.01) were also more likely to think the changes to the legislation would be effective in reducing child drowning deaths (Figure 3).

### 3.2. Child Drowning Deaths

#### 3.2.1. Child Drowning Deaths Relative Risk

**Figure 4:**
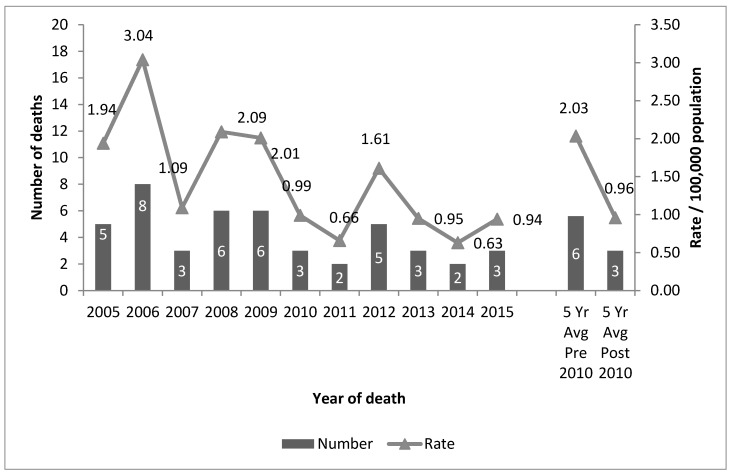
Unintentional fatal drowning in children aged 0–4 years in private swimming pools Queensland, 2005–2015 (*N* = 46).

Between 2005 and 2015 (a period of 11 years), 46 children under the age of five drowned in private swimming pools in Queensland (M = 1.92; SD = 0.85). This represents an annual average age-adjusted rate of 1.45 per 100,000 population and a rate of 7.8 per 10,000 pools with children residing in the residence per annum. In the five years prior to implementation of the legislation (2005–2009) Queensland recorded an annual average of 6 deaths at a rate of 2.03 per 100,000 population. In the five years post implementation (2011–2015), the number of private swimming pool drowning deaths halved, with an annual average of three deaths (a rate of 0.96 per 100,000 population; RR = 2.11). There has been a downward trend in drowning deaths between 2005 and 2015 (y = −0.16x + 2.40; R^2^ = 0.48) (Figure 4).

#### 3.2.2. Child Drowning Deaths Prevalence and Chi Square Analysis

Forty-four (95.7%) drowning deaths occurred in home swimming pools. The remaining 4.3% occurred in portable pools. Slightly more boys than girls were represented in the statistics (54.3% male). Four-fifths (80.5%) of all drowning deaths occurred in children aged between 1 and 2 years. There were no drowning victims under the age of one (Table 3).

Half of all drowning deaths (50.0%) occurred in areas of Queensland classified as major cities, with a further 45.6% taking place in inner and outer regional areas. Home swimming pool drowning deaths in children occurred all year round with the highest proportion occurring in Summer (47.8%), followed by Spring (21.7%). Almost three-quarters (73.9%) of drowning deaths occurred in the afternoon.

A fall into water was the most common activity being undertaken immediately prior to drowning (95.7%), with the remaining children drowning whilst already in the pool (swimming and recreating 4.3%). There were no children who drowned as a result of swimming and recreating in a home swimming pool after the introduction of the legislation (not found to be statistically significant). When examining means of access to the pool area, the most common access point was through a propped open gate (28.3%), followed by a fence that was in disrepair, commonly a gate that didn’t self-close and self-latch (19.6%). In 13.0% of cases, the pool did not have a fence, including the two portable pools (Table 3).

While there was a significant decrease (X^2^(1) = 3.93; *p* < 0.05) in the number of child drowning deaths post the implementation of the new legislation over the study period, there was no statistically significant change across the variables examined compared to the pre legislation period (Table 3).

## 4. Discussion

While the simple and effective solution of pool fencing has been around for many decades [3], the effectiveness of this solution is in the detail. While the latest iteration of pool fencing legislation in Queensland has reduced and maintained lower numbers of home pool-drowning deaths in children under the age of five, the downward trend had started prior to its introduction. This study identified those with home pools were significantly less likely to believe legislative changes would be effective in reducing child-drowning deaths in home swimming pools. 

### 4.1. Trends in Drowning Deaths

Prior to the changes in legislation, children were 2.2 times more likely to drown in home swimming pools in Queensland. There was a downward trend which preceded the introduction of the legislation, potentially due to the high level of both positive and negative publicity which may have increased awareness of the dangers of home swimming pools to young children.

While the numbers were small, the introduction of legislation had no impact on the number of drowning deaths in portable pools. This is unsurprising as many buy very cheap portable pools, which, while only inflated for a small period of time, are not fenced and therefore accessible to all. Novel prevention strategies will be needed to prevent drowning deaths in these types of pools in the future.

In 13.0% of cases, the pool where the child drowned did not have a fence, despite being legally required to have one. This is of concern and raises questions regarding the effectiveness of legislative changes without enforcement. Pool fencing is one of a range of drowning prevention strategies for young children, including supervision, water familiarization, and cardiopulmonary resuscitation (CPR) [28]. We also argue that the pool fence is not only a physical but a psychological barrier and should be treated as such, i.e., discouraging children from climbing the fence. 

Propping the gate open is also a concern and one of the few areas where access to the pool increases post legislation implementation. Effective legislation can be rendered ineffective where the barrier is compromised. This behavioral element will require ongoing strategies to ensure the gate is not left open, and further work is required to understand why people prop the gate open and how this can be addressed. Targeting and educating parents of children under the age of five with home pools on the range of prevention strategies will continue to be required.

### 4.2. The Socio-Ecological Model

While the socio-ecological model proposes five levels, this paper has predominately explored the complexity of the individual’s attitudes to pool fencing legislation. At the individual level, we now know more about attitudes around legislation and pool fencing, however, further work is required to explore people’s knowledge about pool fencing, child drowning, and prevention strategies, as well as individuals’ actual behaviour.

Some of the challenges posed by this research include that those with the least resources (i.e., renters and those on the lowest income) were most likely to see legislation changes as being more effective. They were conversely the groups least likely to enact change and potentially the least likely to be impacted from the changes to legislation. Females were more likely to see the legislative changes as being effective. Generally, females are less likely to be involved in unintentional drowning events [29] and this correlation needs further elucidation.

One of the greatest concerns identified in this study is that people with pools were least likely to think that changes to legislation would be effective. Similar to owning a car, regular maintenance is an essential part of home pool ownership to ensure that it complies with the legislation. This research was unable to say whether the attitudes of pool owners reflects the real-world challenges of constantly ensuring the safety of young children around pools or whether it reflects self-interest and concerns of financial outlay in needing to ensure the pool fence is effective.

The data also suggests that the status of children under the age of five in the household did not impact beliefs about the effectiveness of the legislative changes. We believe that this highlights the importance of promoting legislative change within a drowning prevention lens, rather than as a punitive measure. The evidence shows that pool fencing is effective in reducing child drowning and that the rate of child drowning in Queensland has halved in the five years after legislation was implemented.

Future preventative work needs to take into account specific attitudes around fencing and develop communication tools which target concerns and help to change attitudes and improve behaviour. 

### 4.3. Exposure

Gathering information on exposure is a challenge [30,31,32]. Using representative surveys goes some way towards knowing the true number of home swimming pools in the state of Queensland, however, this is still not an accurate reflection of exposure as we don’t know the type of pool, status of fencing, how often the pool is used, etc. Nor do we know the biases of using a CATI survey to collect the data on home pool ownership.

One measure of exposure to drowning risk is the number of households with a swimming pool. The CATI survey identified that 25.6% of houses in Queensland had a pool. This would equate to 468,543 home pools with a drowning rate of children under the age of five of 0.9 per 100,000 pools per annum. If, however, the number of pools where children under the age of five live is used as the denominator (exposure factor), then the number of pools drops to 53,506 and the rate increases to 7.8 per 100,000 pools per annum, which is an eight-fold increase. This demonstrates that having an accurate calculation of exposure is important for understanding the actual risk. Further work is required to develop better methods of understanding exposure.

### 4.4. Legislation

Most pools on first inspection do not meet the legislative requirements [10,33]. Inspection regimes, even if carried out irregularly, helps to improve the number of pool with compliant barriers [10]. While the barrier is seen as a more effective measure within the Hierarchy of Controls, there is however the ongoing challenge faced by parents in ensuring that young children in their care are kept safe. To help parents understand what is required and to remind them of the strategies available, continuing education will be required to: ensure they supervise their children when in, on, or near water; they check that the pool fence is in good working order; and they create an environment where the child is not allowed to climb the fence [15]. From this survey, there were differing groups where this is going to be more challenging, such as homeowners, people with higher household incomes, people age 45–54, those separated or divorced, and those with a pool at home.

Ensuring that all existing pools are compliant with the latest standard is also a challenge. Work from Queensland found that four-sided pool fencing is more effective than three-sided, and three-sided is more effective than perimeter fencing [34], which has over time been the legislated requirement [8]. If people who lived in residences with a pool were the least likely to see the changes in the legislation as being effective, then they are less likely to bring their pool up to the new standard. The cry of ‘I have never had a problem before why should I change now?’ is all too familiar to those working in injury prevention. Future strategies are required to think about how we might change this attitude.

Recent coronial investigations in Australia have highlighted the risks posed to young children in rental properties with swimming pools, and that often the renters have limited ability to make changes (including fixing the pool fence or gate). In this survey, people who rented were more likely to see the changes to the legislation as being effective in reducing child drowning deaths compared to people who own their own home. This may be due to the challenges they face in ensuring that the premise they live in is safe.

While the number of children drowning in home swimming pools has decreased, we believe there is still room for improvement and a need to ensure those who own a pool take this responsibility seriously. This study highlights groups of people who should be targeted in future safety campaigns.

## 5. Limitations

To explore differences between demographic groups, level of effectiveness was treated as a continuous variable (Figure 1). It is unclear whether people treat the differences as you move between the categories as being equal. For example, does a person see the difference between ‘effective’ and ‘very effective’ the same as between ‘neither effective or ineffective’ and ‘effective’. Caution needs to be used when interpreting Figure 1.

The authors used two time periods (2005–2009 and 2011–2015) with a gap of one year (2010) to see if there was a difference pre and post the introduction of the legislation. However, we note there was a period of five years written into the legislation to allow home pool owners to bring existing swimming pools (i.e., built prior to 1 December 2009) up to the requirements of the 2009 legislation, unless they sold or rented the property, in which case, at that point in time, the pool needed to be brought up to the 2009 legislation. Until the end of the grace period, there would still be pools that would not comply with the 2009 legislation. 

Population-based pool numbers (i.e., the number of households per pool in Queensland) are based on raw responses to the survey, however we note variations within these populations as to who owns a pool and as such there is likely to be variation in the number of pools depending on age of household owners, presence of children, ownership of home, and income and education levels. This is the first time that sociodemographic variables have been used to understand home pool ownership and further work is required to explore the impact of these variables on home ownership and drowning. It is also unclear if using a CATI survey impacts on the likelihood of home pool ownership (negatively/positively) and thus as with all survey data, care should be taken in interpreting the results. 

Care should be exercised when interpreting the chi square analysis where there are small numbers. The response rate to the CATI survey was very low (31.9%) which may have introduced response bias into the results.

## 6. Conclusions

The introduction of legislation has seen a decrease in the number of children drowning in home swimming pools. While at the introduction of this legislation pool owners were less likely to think tightening the legislation would be effective, this has not been reflected in the number of child drowning deaths in home swimming pools. There is still much work to be undertaken to convince people that changes in future pool fencing legislation are beneficial, particularly pool owners. Drowning prevention agencies have more work to do to ensure that the most vulnerable (young children in houses with swimming pools) are protected.

## Figures and Tables

**Table 1:** Computer Assisted Telephone Instrument (CATI) survey responses by pool ownership yes/no by sex, age group, born in Australia, years of education, household income level, child under the age of five, child under the age of 18 in the household, household ownership, and marital status, Queensland, 2011 (*N* = 1263).

	Total		Pool Ownership—Yes		Pool Ownership—No		Degrees of Freedom, X^2^ Analysis Comparing Pool Ownership Yes to Pool Ownership No (*p*-Value)
	N	%	N	%	N	%	
	1263	100.0	324	25.7	939	74.3	
**Sex**							
Male	632	50.0	163	25.8	469	74.2	df = 1, 0.01 (*p* = 0.91)
Female	631	50.0	161	25.5	470	74.5	
**Age Group**							
18–34 years	175	13.9	32	18.3	143	81.7	df = 1, 5.74 (*p* = 0.02)
35–44 years	202	16.0	66	32.7	136	67.3	df = 1, 6.29 (*p* = 0.01)
45–54 years	241	19.1	80	33.2	161	66.8	df = 1, 8.99 (*p* < 0.01)
55+ years	635	50.3	143	22.5	492	77.5	df = 1, 6.49 (*p* = 0.01)
No response	10	0.8	3	0.9	7	0.7	-
**Born in Australia**							
Yes	980	77.6	243	24.8	737	75.2	df = 1, 1.69 (*p* = 0.19)
No	283	22.4	81	28.6	202	71.4	
**Years of Education**							
1–10 years	307	24.3	46	15.0	261	85.0	df = 1, 25.12 (*p* < 0.001)
11–12 years	283	22.4	82	29.0	201	71.0	df = 1, 1.85 (*p* = 0.17)
13–14 years	138	10.9	39	28.3	99	71.7	df = 1, 0.47 (*p* = 0.50)
15+ years	521	41.3	156	29.9	365	70.1	df = 1, 7.77 (*p* = 0.01)
Don’t know/No response	14	1.1	1	7.1	13	92.9	-
**Household Income Level**							
Nil–$26,000	145	11.5	27	18.6	118	81.4	df = 1, 5.21 (*p* = 0.02)
$26,001–$52,000	161	12.7	24	14.9	137	85.1	df = 1, 13.21 (*p* < 0.001)
$52,001–$100,000	206	16.3	48	23.3	158	76.7	df = 1, 1.17 (*p* = 0.28)
Greater than $100,000	287	22.7	110	38.3	177	61.7	df = 1, 34.34 (*p* < 0.001)
Don’t know/No response	464	36.7	115	24.8	349	75.2	-
**Have a Child Under the Age of Five**							
Yes	154	12.2	37	24.0	117	76.0	df = 1, 0.24 (*p* = 0.63)
No	1109	87.8	287	25.9	822	74.1	
**Child Under the Age of 18 in the Household**							
Yes	417	33.0	139	33.3	278	66.7	df = 1, 19.48 (*p* < 0.001)
No	844	66.8	184	21.8	660	78.2	
No response	2	0.2	1	50.0	1	50.0	-
**Household Ownership**							
Rent	210	16.6	20	9.5	190	90.5	df = 1, 34.13 (*p* < 0.001)
Own	1038	82.2	299	28.8	739	71.2	
No response	15	1.2	5	33.3	10	66.7	-
**Marital Status**							
Single/Widowed/Divorced/Separated	326	25.8	62	19.0	264	81.0	df = 1, 10.26 (*p* = 0.001)
Married/De facto	935	74.0	262	28.0	673	72.0	
No response	2	0.2	0	0.0	2	100.0	-

**Table 2:** CATI survey responses by pool fencing effective/ineffective by sex, age group, born in Australia, years of education, household income level, child under the age of five, child under the age of 18 in the household, household ownership, and marital status, Queensland, 2011 (*N* = 1263).

	Total		Pool Fencing Effective		No Response		Pool Fencing Ineffective		Degrees of Freedom, X^2^ Analysis Comparing Pool Fencing Effective to Pool Fencing Ineffective (*p*-Value)
	N	%	N	%	N	%	N	%	
	1263	100.0	726	57.5	38	3.0	499	39.5	
**Sex**									
Male	632	50.0	345	54.6	25	4.0	262	41.5	df = 1, 2.94 (*p* = 0.09)
Female	631	50.0	381	60.4	13	2.1	237	37.6	
**Age Group**									
18–34 years	175	13.9	109	62.3	10	5.7	56	32.0	df = 3, 8.04 (*p* = 0.45)
35–44 years	202	16.0	116	57.4	8	4.0	78	38.6	
45–54 years	241	19.1	123	51.0	6	2.5	112	46.5	
55+ years	635	50.3	374	58.9	13	2.0	248	39.1	
No response	10	0.8	4	40.0	5	50.0	1	10.0	-
**Born in Australia**									
Yes	980	77.6	571	58.3	28	2.9	381	38.9	df = 1, 0.90 (*p* = 0.34)
No	283	22.4	155	54.8	10	3.5	118	41.7	
**Years of Education**									
1–10 years	307	24.3	189	61.6	5	1.6	113	36.8	df = 3, 4.80 (*p* = 0.19)
11–12 years	283	22.4	163	57.6	5	1.8	115	40.6	
13–14 years	138	10.9	69	50.0	4	2.9	65	47.1	
15+ years	521	41.3	297	57.0	23	4.4	201	38.6	
Don’t know/No response	14	1.1	8	57.1	1	7.1	5	35.7	-
**Household Income Level**									
Nil–$26,000	145	11.5	96	66.2	3	2.1	46	31.7	df = 1, 5.77 (*p* = 0.02)
$26,001–$52,000	161	12.7	96	59.6	2	1.2	63	39.1	df = 1, 0.25 (*p* = 0.62)
$52,001–$100,000	206	16.3	118	57.3	7	3.4	81	39.3	df = 1, 0.05 (*p* = 0.83)
Greater than $100,000	287	22.7	145	50.5	11	3.8	131	45.6	df = 1, 6.57 (*p* = 0.01)
Don’t know/No response	464	36.7	271	58.4	15	3.2	178	38.4	-
**Have a Child Under the Age of Five**									
Yes	154	12.2	93	60.4	5	3.2	56	36.4	df = 1, 0.70 (*p* = 0.40)
No	1109	87.8	633	57.1	33	3.0	443	39.9	
**Children Under the Age of 18 in the Household**									
Yes	417	33.0	246	59.0	15	3.6	156	37.4	df = 1, 0.91 (*p* = 0.34)
No	844	66.8	479	56.8	23	2.7	342	40.5	
No response	2	0.2	1	50.0	0	0.0	1	50.0	-
**Household Ownership**									
Rent	210	16.6	137	65.2	8	3.8	65	31.0	df = 1, 7.51 (*p* < 0.01)
Own	1038	82.2	579	55.8	30	2.9	429	41.3	
No response	15	1.2	10	66.7	0	0.0	5	33.3	-
**Marital Status**									
Single/Widowed/Divorced/Separated	326	25.8	213	65.3	8	2.5	105	32.2	df = 1, 10.56 (*p* = 0.001)
Married/De facto	935	74.0	512	54.8	30	3.2	393	42.0	
No response	2	0.2	1	50.0	0	0.0	1	50.0	-
**Pool Ownership**									
Yes	324	25.7	144	44.4	9	2.8	171	52.8	df = 1, 32.25 (*p* < 0.001)
No	939	74.3	582	62.0	29	3.1	328	34.9	

**Table 3:** Drowning deaths of children aged 0–4 years in private swimming pools by sex, single year of age, pool type, remoteness classification of drowning incident location, season of drowning incident, time of day of drowning incident, activity immediately prior to drowning, and point of entry to pool, Queensland, 2005–2015 (*N* = 46).

	Total		Before Implementation (2005–2009)		Implementation Year (2010)		After Implementation (2011–2015)		Degrees of Freedom, X^2^ Analysis Comparing before and after Implementation (*p*-Value)
	N	%	N	%	N	%	N	%	
Total	46	100.0	28	60.9	3	6.5	15	32.6	df = 1, 3.93 (*p* < 0.05)
**Sex**									
Male	25	54.3	13	52.0	1	4.0	11	44.0	df = 1, 2.87 (*p* = 0.09)
Female	21	45.7	15	71.4	2	9.5	4	19.0	
**Single Year of Age**									
0 year	0	0.0	0	0.0	0	0.0	0	0.0	df = 3, 4.04 (*p* = 0.13)
1 year	17	37.0	8	47.1	0	0.0	9	52.9	
2 years	20	43.5	13	65.0	3	15.0	4	20.0	
3–4 years	9	19.5	7	77.8	0	0.0	2	22.2	
**Remoteness Classification of Drowning Incident Location**									
Major Cities	23	50.0	15	65.2	1	4.3	7	30.4	df = 3, 1.74 (*p* = 0.63)
Inner Regional	10	21.7	5	50.0	0	0.0	5	50.0	
Outer Regional	11	23.9	7	63.6	1	9.1	3	27.3	
Remote & Very Remote	2	4.3.0	1	50.0	1	50.0	0	0.0	
**Season of Drowning Incident**									
Summer	22	47.8	11	50.0	2	9.1	9	40.9	df = 3, 2.34 (*p* = 0.51)
Autumn	7	15.2	5	71.4	0	0.0	2	28.6	
Winter	7	15.2	6	85.7	0	0.0	1	14.3	
Spring	10	21.7	6	60.0	1	10.0	3	30.0	
Time of Day of Drowning Incident *									
Morning	9	20.0	5	55.6	2	22.2	2	22.2	df = 2, 0.34 (*p* = 0.84)
Afternoon	34	75.6	21	61.8	1	2.9	12	35.3	
Evening & Early Morning	2	4.4	1	50.0	0	0.0	1	50.0	
**Point of Entry**									
No fence	6	13.0	3	50.0	1	16.7	2	33.3	df = 6, 12.79 (*p* = 0.05)
Gate propped open	13	28.3	4	30.8	0	0.0	9	69.2	
Disrepair—faulty gate, climbed under, loose panels etc.	9	19.6	7	77.8	0	0.0	2	22.2	
Child climbed over fence	4	8.7	3	75.0	1	25.0	0	0.0	
Child already in pool area	3	6.5	2	66.7	0	0.0	1	33.3	
No isolation fencing, child accessed pool through door, window etc.	4	8.7	4	100.0	0	0.0	0	0.0	
Child used an object to open gate	3	6.5	3	100.0	0	0.0	0	0.0	
Unknown	4	8.7	2	50.0	1	25.0	1	25.0	

Note: Please note that no results were statistically significant. * There was one case where time of day of drowning incident was not known.
